# A multidimensional nomogram for clinical risk stratification of bloodstream infection and mortality in hematologic malignancies

**DOI:** 10.3389/fcimb.2026.1834653

**Published:** 2026-08-27

**Authors:** Lingqiong Lan, Shaozhen Chen, Haojie Zhu, Jixin Fan, Hui Kong, Jinhua Ren, Jianda Hu, Ting Yang

**Affiliations:** 1 The Second Department of Hematology, National Regional Medical Center, Binhai Campus of the First Affiliated Hospital, Fujian Medical University, Fuzhou, China; 2 Department of Hematology, Longyan Second Hospital, Longyan, China; 3 Fujian Medical University Cancer Hospital, Fujian Cancer Hospital, Fuzhou, China; 4 Department of Hematology, Fujian Institute of Hematology, Fujian Provincial Key Laboratory of Hematology, Fujian Medical University Union Hospital, Fuzhou, China; 5 The Second Department of Hematology, The First Affiliated Hospital, Fujian Medical University, Fuzhou, China; 6 The Second Affiliated Hospital of Fujian Medical University, Quanzhou, China; 7 Institute of Precision Medicine, Fujian Medical University, Fuzhou, China

**Keywords:** bloodstream infection, hematological malignancies, internal validation, nomogram, prediction model

## Abstract

**Background:**

Bloodstream infections (BSIs) are serious complications in patients with hematologic malignancies (HMs) and are associated with substantial morbidity and mortality. Effective risk stratification is crucial for optimizing empirical antimicrobial decisions. This study aimed to develop and internally validate nomograms for predicting BSI occurrence and post-BSI mortality among HM patients.

**Methods:**

This retrospective cohort study enrolled 3,014 HM patients, including 725 with BSI. The cohort was randomly divided into derivation and internal validation cohorts at a 6:4 ratio. The BSI occurrence model was developed using multivariable logistic regression, while the post-BSI mortality prediction model among patients with BSI was developed using Cox regression. Model performance was evaluated based on discrimination, calibration, and decision curve analysis.

**Results:**

The BSI occurrence model demonstrated acceptable discrimination, with area under the receiver operating characteristic curve (AUC) values of 0.775 in the derivation cohort and 0.761 in the internal validation cohort. The final nomogram incorporated 12 variables, among which neutrophil count ≤0.5 × 10^9^/L received a relatively high point allocation. The post-BSI mortality prediction nomogram showed moderate discrimination, with C-indices of 0.721 and 0.713 in the derivation and internal validation cohorts, respectively. Nine variables were included in the final post-BSI mortality model, and polymicrobial BSI received a relatively high point allocation within the nomogram. Both models demonstrated acceptable calibration and potential clinical utility based on decision curve analysis. The BSI occurrence model was further assessed in a small independent cohort, whereas the mortality model requires additional external validation.

**Conclusions:**

The BSI occurrence model showed acceptable discrimination in the derivation and internal validation cohorts and was preliminarily assessed in an independent cohort. The post-BSI mortality model showed moderate discrimination in the internal validation cohort and requires further evaluation in larger and more representative populations.

## Introduction

Bloodstream infections (BSIs) represent a persistent and catastrophic complication in patients with hematological malignancies (HM), serving as a primary driver of non-relapse mortality and escalating healthcare expenditures globally (Z. Li et al., 2025; Zha et al., 2025). Despite significant advancements in antineoplastic therapies and supportive care, BSIs remain a formidable challenge in this population, reported incidence rates ranging from 20.8% to 24.1% (Chen et al., 2021; El et al., 2024; Okamoto et al., 2021), associated with high mortality rates (23.6%-29.0%) (Chen et al., 2021; Wang et al., 2023). The epidemiological landscape of these infections has evolved rapidly in recent years, characterized by a resurgence of Gram-negative bacteria (GNB) and a precipitous rise in multidrug-resistant (MDR) organisms, including carbapenem-resistant Enterobacteriaceae (Capsoni et al., 2025; S. Chen et al., 2025; Zha et al., 2025). Infection with these resistant pathogens is associated with limited therapeutic options and can lead to mortality rates exceeding 50% if septic shock supervenes or effective treatment is delayed (Park et al., 2024).

The survival of HM patients with BSIs is contingent upon the timely administration of appropriate antimicrobial therapy, a concept widely recognized as the “golden hour” (Gretland et al., 2025). Recent systematic reviews and clinical studies have confirmed a linear relationship between delays in antimicrobial initiation and increased mortality in septic patients (Liu et al., 2017; Tang et al., 2024). However, the clinical presentation of febrile neutropenia is often non-specific, creating a profound diagnostic dilemma. Current standard risk stratification tools, such as the Multinational Association for Supportive Care in Cancer risk index and the Clinical Index of Stable Febrile Neutropenia, were primarily designed to identify low-risk patients suitable for outpatient management (Alpar and Yılmaz, 2025). Emerging evidence from 2024 and 2025 validation studies indicates that these scoring systems exhibit suboptimal sensitivity and specificity when applied to high-risk hospitalized HM cohorts, often failing to adequately stratify patients with profound neutropenia or predicting rapid deterioration due to MDR pathogens (Alpar and Yılmaz, 2025; Kanter et al., 2025; Shakinah et al., 2024). Furthermore, reliance on culture-based diagnosis entails an inherent time delay in obtaining definitive pathogen identification and antimicrobial susceptibility results (Lamy et al., 2020), a critical window during which inappropriate empirical therapy can result in irreversible adverse outcomes (Allel et al., 2024).

Given these limitations, there is an urgent imperative to transition from reactive management strategies to proactive, precision-based risk stratification. The development of clinical prediction models, specifically nomograms, has gained traction as a vital tool for personalized medicine (Guo et al., 2025; Wang et al., 2023). Unlike traditional analyses that retrospectively identify isolated risk factors, predictive nomograms integrate multiple continuous and categorical variables—such as biomarkers (Y. Chen et al., 2025), catheter history (Guo et al., 2025), and treatment intensity—to generate an individualized numerical probability of BSI occurrence and prognosis at the bedside (Wang et al., 2023). While recent efforts have utilized machine learning to predict sepsis outcomes (Q. Li et al., 2025; Park et al., 2025), few studies have established practical, clinically interpretable nomograms derived from large-scale datasets specifically tailored to the unique physiological landscape of hematological malignancies (Chen et al., 2021; Garcia-Vidal et al., 2018).

The source cohort was previously used in an epidemiological study describing the incidence, microbiological characteristics, and factors associated with BSI occurrence and mortality (Chen et al., 2021). That study focused on the incidence, microbiological characteristics, antimicrobial resistance patterns, and factors associated with BSI occurrence and post-BSI mortality. The present study represents a secondary prediction-model analysis using the same source cohort, with the aim of developing and evaluating clinically interpretable nomograms for individualized risk stratification.

## Methods

### Setting and study design

This study received ethical approval from the Institutional Review Board of Fujian Medical University Union Hospital (2020KY019), with the requirement for informed consent waived due to its retrospective design. We conducted a retrospective analysis of patients with hematologic malignancies who underwent blood culture testing at this major teaching hospital in Fuzhou, China. The study period spanned from January 1, 2013, to December 31, 2016. The source population overlapped with the cohort described in our previous epidemiological publication. The current analysis was conducted with a distinct prediction-focused objective and should be interpreted as a model-development study rather than a new epidemiological cohort analysis. We followed the following inclusion criteria to screen patients: (1) patient diagnosed with HM; (2) available BC testing data. The exclusion criteria were as follows: (1) patient diagnosed with non-HM; (2) recurrent infections were excluded, and only the first BSI episode per patient was included in our analysis. BSI and non-BSI subgroups were compared to identify the predictors of BSI occurrence. Meanwhile, the post-BSI mortality prediction model was developed to predict all-cause mortality after BSI occurrence.

### Data collection

We collected relevant demographic and clinical data from the hospital’s electronic medical record system. The retrieved information encompassed patient gender, age, primary hematologic diagnosis and its status (remission/non-remission), comorbidities (including diabetes), hospitalization duration, chemotherapy history, laboratory parameters, details of any co-infections, antimicrobial therapies administered, and clinical outcomes.

### Bacterial isolates

Blood culture (BC) specimens were processed according to the laboratory’s standard operating procedures (National Health Commission Of China, 2017). Briefly, samples were incubated using the automated BACT/ALERT 3D system (BioMerieux, France) at 37 °C under 5% CO_2_ atmosphere for up to 7 days. Microbial growth from positive cultures was subsequently isolated, and all bacterial isolates were identified using the VITEK2 automated platform (BioMerieux, France). Antimicrobial susceptibility testing was performed following the prevailing Clinical and Laboratory Standards Institute (CLSI) guidelines (Clinical And Laboratory Standards Institute, 2023).

### Definitions

There are no widely used criteria for the diagnosis of BSI in patients with HM receiving chemotherapy. We used the definitions proposed by Kameda et al. (Kameda et al., 2016) to define a definite or a probable BSI. Briefly, a “definite BSI” was defined as the isolation from at least one BC of a bacterial or fungal pathogen other than common skin contaminants. For common skin contaminants such as *Diphtheroids*, *Bacillus spp*, *Propionibacterium spp*, *coagulase-negative Staphylococci*, *Viridans Streptococci*, *Aerococcus spp*, and *Micrococcus spp* (Hossain et al., 2016; Richter et al., 2002), detection in 2 or more separate blood cultures was required for a definite BSI diagnosis. Other BC positive cases were defined as probable BSI.

A BSI was considered polymicrobial if two or more distinct microbial species were concurrently isolated from a single BC set (Yo et al., 2019). We defined neutropenia according to standard oncologic criteria (Boccia et al., 2022): an absolute neutrophil count below 0.5×10^9^/L, or a count below 1.0×10^9^/L that was anticipated to fall to 0.5×10^9^/L or lower within the subsequent 48-hour period.

The index time point was defined as the clinical evaluation associated with BC collection. For antimicrobial exposure, the recorded variable represented antimicrobial agents already prescribed and documented at the time of the index BC collection. Antimicrobial agents initiated or added after microbiological confirmation of BSI were not included in this predictor. Hospital length of stay was treated as a dynamic inpatient variable reflecting the accumulated duration and complexity of the hospital course associated with the index BC evaluation rather than an admission-level baseline characteristic. The outcome of the post-BSI mortality prediction model was all-cause mortality after BSI occurrence. Survival probabilities were evaluated at predefined time points of 7 days, 14 days, 30 days, and 1 year after BSI occurrence.

### Statistical analyses

Statistical analyses were performed using R software (version 4.0.3). Missing data were handled using multiple imputation with the mice package to reduce potential bias and loss of statistical efficiency associated with complete-case analysis. Variables with more than 50% missing values were excluded before imputation. The entire cohort was randomly divided into a derivation cohort and an internal validation cohort at a ratio of 6:4. The BSI occurrence prediction model was developed using the entire cohort of 3,014 patients with hematologic malignancies undergoing blood culture evaluation, including 1,809 patients in the derivation cohort and 1,205 patients in the internal validation cohort (**Supplementary Table 1**). Among patients with confirmed BSI (n=725), a separate post-BSI mortality prediction model was developed using the BSI subgroup as an independent analytical population. These patients were divided into a mortality model derivation cohort (n=435) and an internal validation cohort (n=290), maintaining an approximately 6:4 allocation ratio (**Supplementary Table 2**). Therefore, the analytical populations and cohort allocation strategies were defined separately according to the clinical objectives of the two prediction models. Model development and variable selection were performed in the derivation cohort, whereas model discrimination, calibration, and clinical utility were subsequently assessed in both cohorts. This internal validation was based on a single random derivation-validation split. Bootstrap resampling and k-fold cross-validation were not performed.

For the BSI occurrence model, univariable and multivariable logistic regression analyses were performed in the derivation cohort to evaluate candidate variables for model development. Variables showing potential associations in univariable analyses were screened for subsequent multivariable modeling. Model selection was subsequently performed using bidirectional stepwise regression based on the Akaike information criterion (AIC), and the model with the lowest AIC value was selected as the final model. For the BSI occurrence model, variable coding and regression coefficients were additionally provided in **Supplementary Table 3**.

For the post-BSI mortality prediction model among patients with BSI, univariable and multivariable Cox proportional hazards regression analyses were performed in the derivation cohort. Candidate variables meeting the prespecified screening criterion were further evaluated using bidirectional stepwise regression based on AIC, and the model with the lowest AIC value was selected as the final prognostic model. The final post-BSI mortality prediction model was specified using the selected independent predictors. Variable coding and regression coefficients are provided in **Supplementary Table 4**.

Variables retained in the final AIC-selected models were considered model predictors rather than causal factors or individually significant risk factors. Inclusion in the final models did not necessarily require that each individual multivariable coefficient reached statistical significance (P < 0.05). To ensure consistency and reproducibility, categorical variables were coded using the same reference categories in both regression analyses and nomogram construction.

Model performance was evaluated in both the derivation and internal validation cohorts. Discrimination of the BSI occurrence model was assessed using receiver operating characteristic (ROC) curve analysis and the area under the curve (AUC). The discrimination of the post-BSI mortality prediction model was evaluated using Harrell’s concordance index (C-index). Kaplan–Meier survival curves were generated according to nomogram-derived risk groups to assess risk stratification performance.

Model calibration was evaluated using calibration plots comparing predicted and observed outcomes. The Hosmer–Lemeshow goodness-of-fit test was additionally used for calibration assessment of the logistic regression model. Clinical utility was evaluated using decision curve analysis (DCA) with the rmda package.

An independent cohort of acute leukemia patients undergoing hematopoietic stem cell transplantation was used for preliminary assessment of the BSI occurrence model. Due to the limited number and imbalanced distribution of mortality events, this independent cohort was not used for external validation of the post-BSI mortality prediction model.

A two-sided P value <0.05 was considered statistically significant.

## Results

### Cohort partition and baseline characteristics for model development

To develop and validate the prediction models, the entire cohort of 3,014 patients was randomly divided into a derivation cohort (n=1,809) and an internal validation set (n=1,205) at a 6:4 ratio. The baseline characteristics of the derivation and validation cohorts were comparable, except for gender distribution (**Supplementary Table 1**), ensuring the validity of the subsequent modeling process.

### Development of the nomogram for predicting BSI occurrence

**Figure 1:**
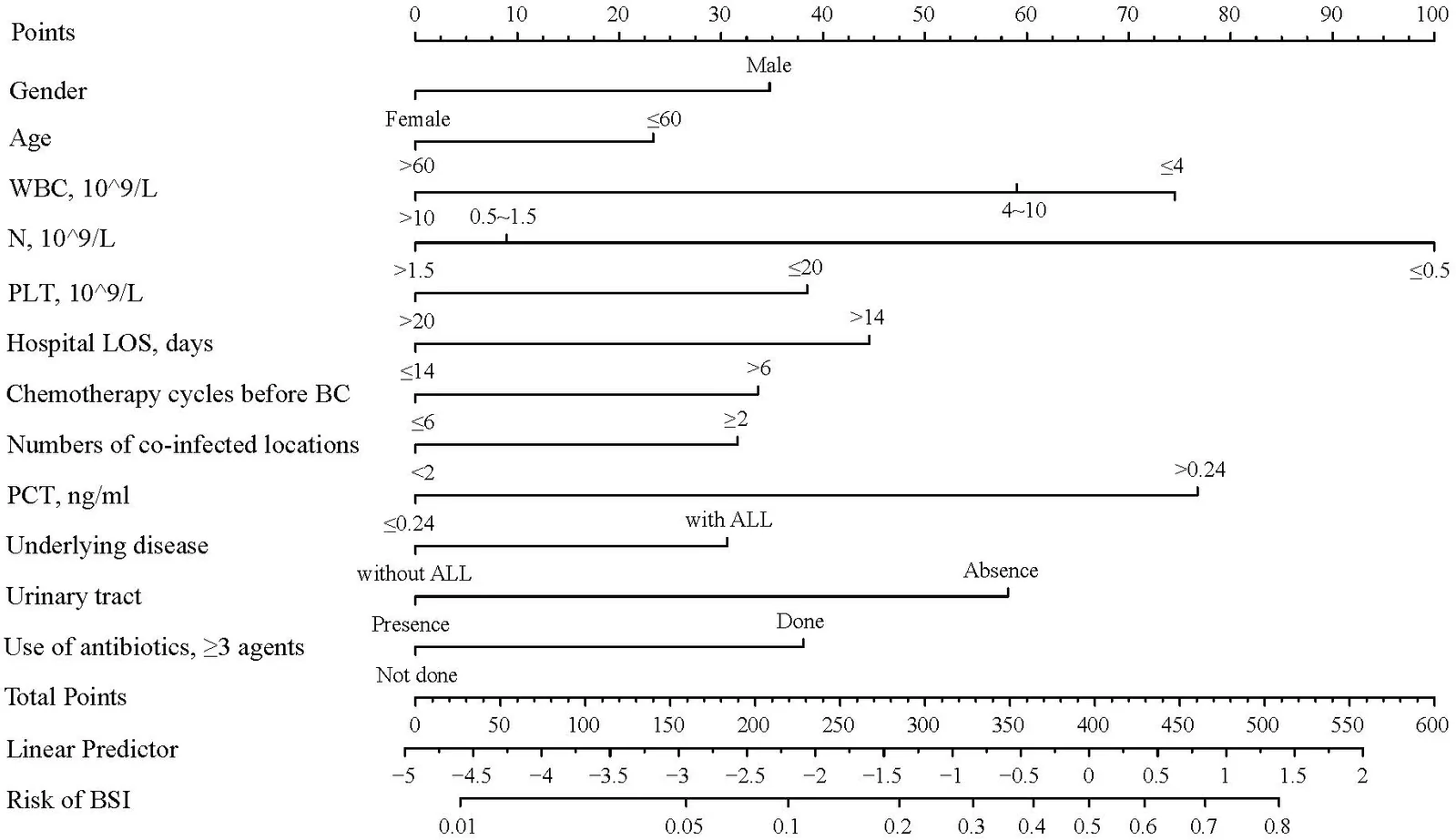
Nomogram for predicting the probability of bloodstream infection occurrence in patients with hematologic malignancies. WBC, white blood cell count; N, neutrophilic count; PLT, platelet count; LOS, length of stay; PCT, procalcitonin; BC, blood culture.

Candidate variables associated with BSI occurrence were initially evaluated using univariable and multivariable logistic regression analyses (**Table 1**). Based on these candidate variables, further model selection was performed using the AIC. The final AIC-selected model retained 12 variables that jointly contributed to prediction of BSI status, including male gender, age < 60 years, diagnosis of ALL, hospital length of stay (LOS) >14 days, white blood cell count (WBC) ≤ 4×10^9^/L, neutrophil count (N) ≤ 0.5 ×10^9^/L, platelet count (PLT) ≤ 20 ×10^9^/L, elevated procalcitonin (PCT > 0.24 ng/mL), > 6 prior chemotherapy cycles, multi-site co-infection, concurrent infection in specific regions (oral, gastrointestinal, perirectal, or urinary tract), and use of ≥3 antibiotics. This model was transformed into a nomogram for clinical use (**Figure 1**). Among the variables included in the nomogram, N and PLT received relatively higher point allocations. Using the Youden index (0.41), the optimal nomogram score cutoff was determined to be 307 points, stratifying patients into higher (score ≥307) and lower (score <307) risk groups for BSI occurrence.

**Table 1:** Candidate predictors associated with BSI occurrence in the derivation cohort.

Variable	Coding	Univariate analysis		Multivariate analysis	
		OR (95% CI)	P-value	OR (95% CI)	P-value
Gender	Male vs Female	1.496 (1.199, 1.867)	<0.001	1.539 (1.202, 1.971)	<0.001
Age	>60 vs ≤60	0.664 (0.487, 0.906)	<0.010	0.757 (0.533, 1.074)	0.119
Diagnosis with AML	Yes vs No	0.831 (0.671, 1.03)	0.090		
Diagnosis with ALL	Yes vs No	2.111 (1.641, 2.715)	<0.001	1.462 (1.098, 1.947)	<0.010
Disease status	Non-remission vs Remission	1.003 (0.774, 1.298)	0.984		
Hospital LOS, days	>14 vs ≤14	1.955 (1.55, 2.465)	<0.001	1.629 (1.224, 2.168)	<0.001
WBC, 10^9/L	4~10 vs ≤4	0.304 (0.203, 0.455)	<0.001	1.221 (0.665, 2.242)	0.519
WBC, 10^9/L	>10 vs ≤4	0.18 (0.107, 0.304)	<0.001	0.485 (0.248, 0.947)	0.034
N, 10^9/L	0.5~1.5 vs ≤0.5	0.274 (0.169, 0.444)	<0.001	0.339 (0.2, 0.574)	<0.001
N, 10^9/L	>1.5 vs ≤0.5	0.178 (0.127, 0.248)	<0.001	0.309 (0.178, 0.537)	<0.001
HB, g/L	>60 vs ≤60	0.551 (0.444, 0.684)	<0.001	0.872 (0.678, 1.122)	0.287
PLT, 10^9/L	>20 vs ≤20	0.389 (0.313, 0.485)	<0.001	0.636 (0.494, 0.818)	<0.001
PCT, ng/ml	>0.24 vs ≤0.24	2.718 (2.172, 3.401)	<0.001	2.602 (2.032, 3.332)	<0.001
Duration of neutropenia before BC, days	>7 vs ≤7	2.387 (1.829, 3.115)	<0.001	1.188 (0.866, 1.63)	0.286
Chemotherapy cycles before BC	>6 vs ≤6	1.518 (1.176, 1.96)	<0.010	1.535 (1.149, 2.049)	<0.010
Numbers of co-infected locations	≥2 vs <2	2.324 (1.682, 3.212)	<0.001	1.379 (0.852, 2.231)	0.191
Co-infections					
Oral	Presence vs Absence	1.616 (1.257, 2.078)	<0.001	1.047 (0.755, 1.452)	0.783
Respiratory	Presence vs Absence	0.996 (0.764, 1.298)	0.975		
Gastrointestinal	Presence vs Absence	1.617 (1.232, 2.124)	<0.001	1.02 (0.726, 1.435)	0.907
Skin and soft tissue	Presence vs Absence	1.262 (0.86, 1.853)	0.235		
Perirectal	Presence vs Absence	1.811 (1.298, 2.527)	<0.001	1.112 (0.753, 1.642)	0.593
Urinary tract	Presence vs Absence	2.363 (1.328, 4.205)	<0.010	2.112 (1.088, 4.1)	0.027
Other	Presence vs Absence	1.397 (0.736, 2.65)	0.306		
Diabetes mellitus	Presence vs Absence	1.073 (0.761, 1.514)	0.687		
Use of antibiotics, ≥3 agents	Done vs Not done	2.636 (2.003, 3.469)	<0.001	1.561 (1.131, 2.154)	<0.010

For categorical variables, the coding direction indicates the comparison with the reference category used in regression analyses. The coding strategy was consistent between regression models and nomograms. BSI, bloodstream infection; AML, acute myeloid leukemia; ALL, acute lymphoblastic leukemia; LOS, length of stay; WBC, white blood cell count; N, neutrophilic count; HB, hemoglobin; PLT, platelet count; PCT, procalcitonin; BC, blood culture.

### Calibration and validation of the nomogram for predicting BSI occurrence

**Figure 2:**
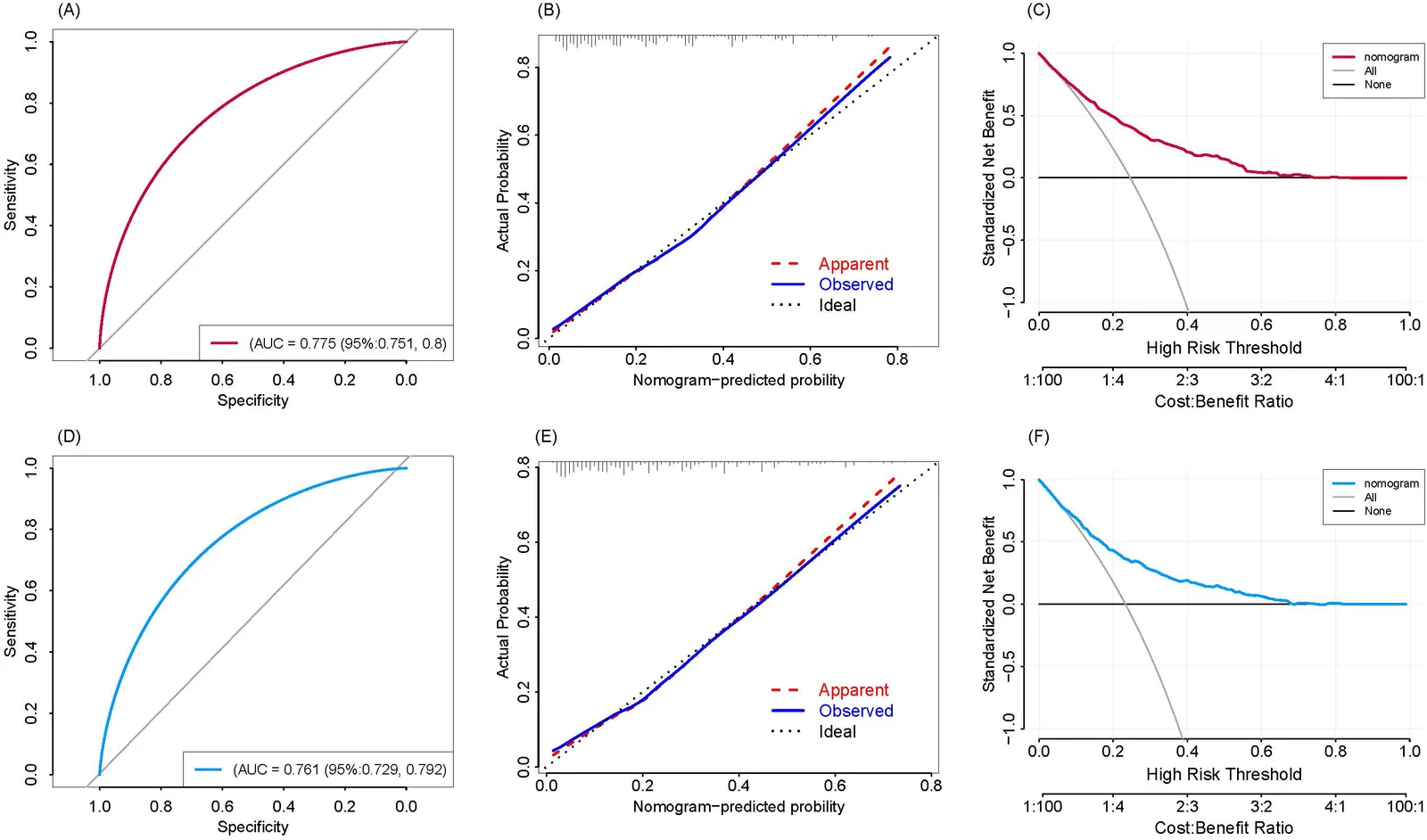
Performance of the BSI occurrence prediction model in the derivation cohort and internal validation cohort. **(A)** The ROC curve and area under the curve (AUC) of the BSI occurrence model in the derivation cohort. The gray solid line indicates no discriminability (AUC = 0.5). **(B)** Calibration curve of the BSI occurrence model in the derivation cohort. The black dotted diagonal line indicates the perfect correspondence between the observation and prediction. **(C)** The Clinical Decision Curve (CDC) of the BSI occurrence model in the derivation cohort. The x-axis represents the range of threshold probabilities, indicating the likelihood at which the model predicts positive cases. The y-axis shows the net benefit, denoting the clinical utility gained by using the model for decision-making. The red line represents the model’s decision curve, while the gray or black line represents the baseline decision (all or none BSI). The area between the curves demonstrates the range of threshold probabilities where the model provides a higher net benefit compared to the baseline strategy. **(D)** ROC curve and AUC of the BSI occurrence model in the internal validation cohort. The gray solid line indicates no discriminability (AUC = 0.5). **(E)** Calibration curve of the BSI occurrence model in the internal validation cohort. The black dotted diagonal line indicates the perfect correspondence between the observation and prediction. **(F)** The CDC analysis of the BSI occurrence model in the internal validation cohort. The x-axis represents the range of threshold probabilities, indicating the likelihood at which the model predicts positive cases. The y-axis shows the net benefit, denoting the clinical utility gained by using the model for decision-making. The blue line represents the model’s decision curve, while the gray or black line represents the baseline decision (all or none BSI). The area between the curves demonstrates the range of threshold probabilities where the model provides a higher net benefit compared to the baseline strategy.

In order to assess the goodness-of-fit of the nomogram for the BSI risk model, we performed the Hosmer-Lemeshow test. The test yielded a chi-square value of 5.19 in the derivation cohort and 11.86 in the validation cohort, respectively. These results indicate that the model fits the data well, as no significant difference was found between the observed and predicted values (both p > 0.05). The occurrence model yielded an AUC of 0.775 (95% CI, 0.75–0.80; **Figure 2A**) in the derivation cohort and 0.761 (95% CI, 0.73–0.79; **Figure 2D**) in the internal validation cohort. As illustrated in **Figures 2B, E**, the calibration plots for the nomogram in both cohorts indicated that the risk of BSI occurrence predicted by the model closely aligned with the actual observed risk. The DCA results demonstrated that the nomogram provided a net benefit across a range of threshold probabilities, indicating that the model offers valuable guidance in clinical decision-making compared to all patients with BC or none with BC (**Figures 2C, F**).

### Development of the nomogram for predicting post-BSI mortality

**Figure 3:**
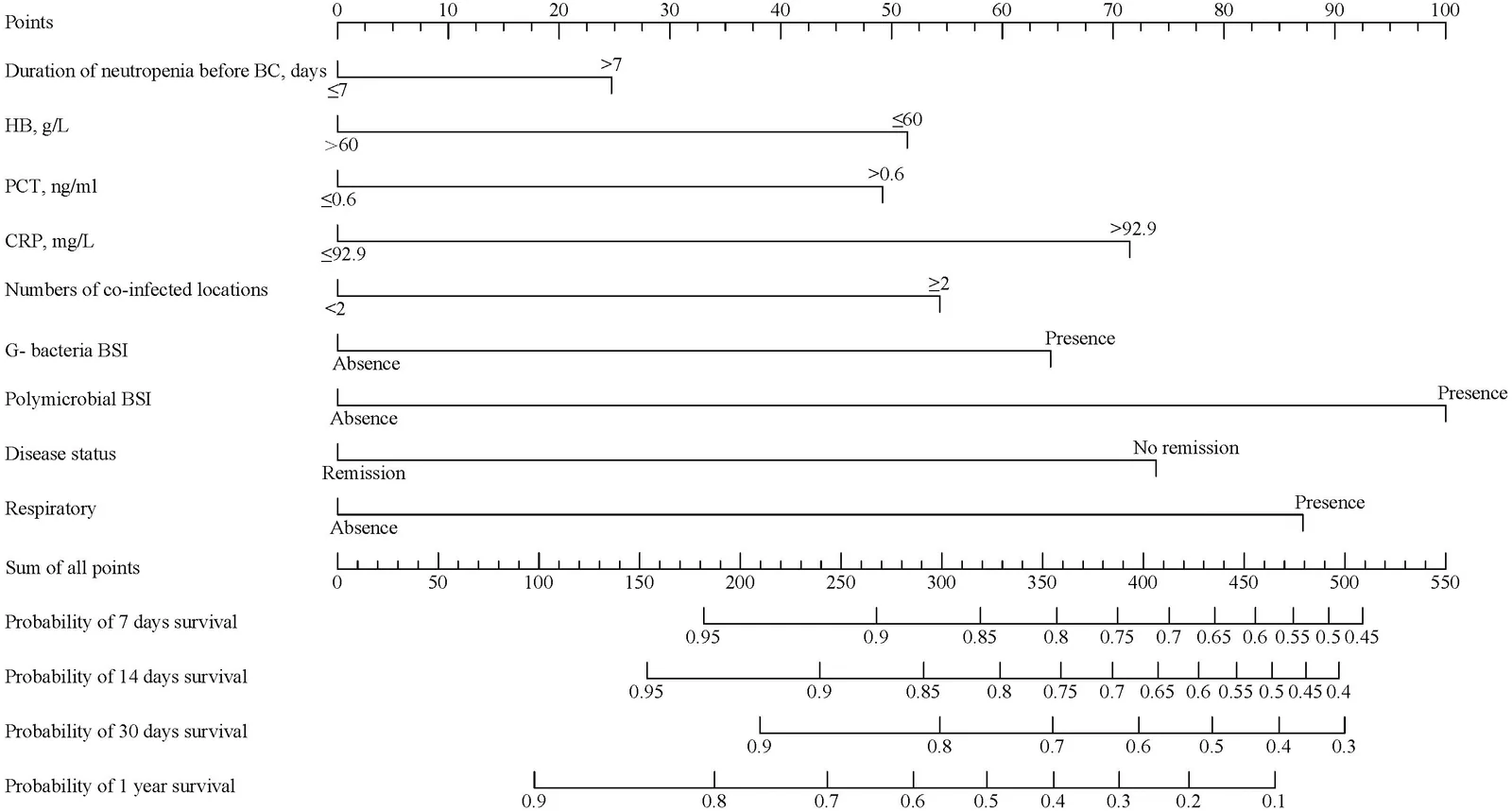
Nomograms for predicting post-BSI mortality probability at predefined time points after BSI occurrence. HB, hemoglobin; PCT, procalcitonin; CRP, C-reactive protein; BC, blood culture; BSI, bloodstream infection; G-, Gram-negative.

**Figure 4:**
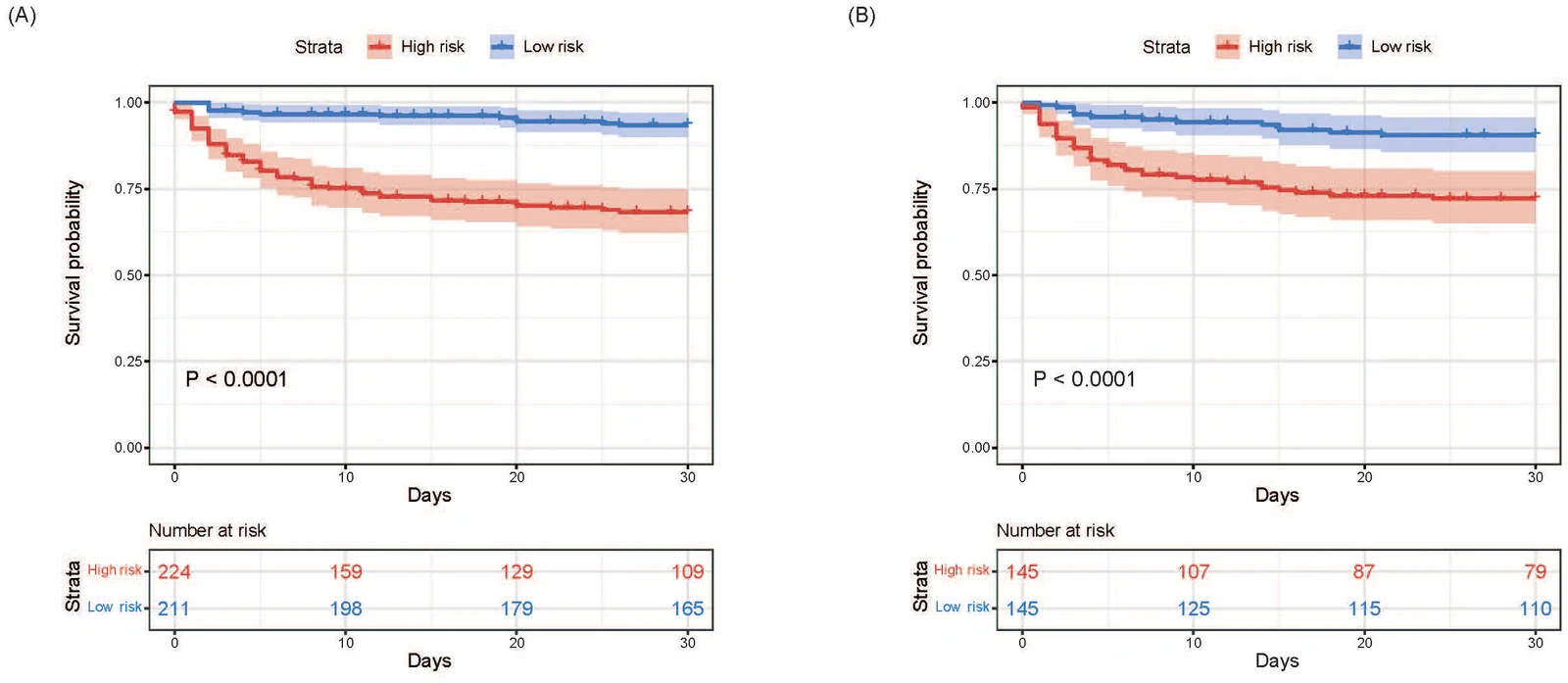
Kaplan-Meier estimates the survival of patients in high or low risk group. **(A)** Kaplan-Meier estimates the survival of BSI patients in the derivation cohort. **(B)** Kaplan-Meier estimates the survival of BSI patients in the internal validation cohort. The high-risk group of patients exhibited reduced probabilities of survival, P<0.0001.

Among patients with BSI, variables associated with mortality were evaluated using univariable and multivariable Cox proportional hazards regression analyses (**Table 2**). Subsequently, AIC-based model selection was performed to establish the final post-BSI mortality prediction model. The final AIC-selected mortality model retained nine model variables: prolonged neutropenia (>7 days) before BC, hemoglobin (HB) ≤ 60 g/L, elevated procalcitonin (>0.6 ng/mL), C-reactive protein (CRP) >92.9 mg/L, multi-site co-infection, GNB bacteremia, polymicrobial BSI, non-remission status, and concurrent respiratory infection. This model was visualized as a nomogram (**Figure 3**). In this nomogram, polymicrobial BSI was assigned a relatively high point score, followed by concurrent respiratory infection and non-remission status. Based on the Youden-derived risk index (2.789), the optimal nomogram score cutoff was determined to be 272 points. This threshold effectively stratified patients into high-risk (score ≥272) and low-risk (score <272) groups, with the high-risk group demonstrating significantly higher 30-day mortality (**Figure 4**).

**Table 2:** Candidate predictors associated with post-BSI mortality among patients with BSI in the derivation cohort.

Variable	Coding	Univariate analysis		Multivariate analysis	
		HR (95%CI)	P-value	HR (95%CI)	P-value
Gender	Male vs Female	1.188 (0.765, 1.844)	0.442		
Age	>60 vs ≤60	0.78 (0.376, 1.618)	0.504		
Diagnosis with AML	Yes vs No	0.638 (0.406, 1.001)	0.051		
Diagnosis with ALL	Yes vs No	1.148 (0.723, 1.823)	0.558		
Disease status	Non-remission vs Remission	2.522 (1.261, 5.045)	0.009	1.744 (0.83, 3.664)	0.142
Diabetes mellitus	Presence vs Absence	0.585 (0.237, 1.446)	0.246		
Hospital LOS, days	>14 vs ≤14	0.846 (0.525, 1.363)	0.492		
WBC, 10^9/L	4~10 vs ≤4	0.942 (0.344, 2.577)	0.908		
WBC, 10^9/L	>10 vs ≤4	0.91 (0.287, 2.887)	0.873		
N, 10^9/L	0.5~1.5 vs ≤0.5	2.214 (0.961, 5.104)	0.062		
N, 10^9/L	>1.5 vs ≤0.5	0.972 (0.422, 2.24)	0.948		
HB, g/L	>60 vs ≤60	0.479 (0.302, 0.761)	0.002	0.592 (0.359, 0.977)	0.040
PLT, 10^9/L	>20 vs ≤20	0.665 (0.415, 1.066)	0.090		
PCT, ng/ml	>0.6 vs ≤0.6	2.354 (1.482, 3.738)	<0.001	2.416 (1.497, 3.898)	<0.001
CRP, mg/L	>92.9 vs ≤92.9	1.773 (1.129, 2.785)	0.013	1.389 (0.872, 2.21)	0.166
Duration of neutropenia before BC, days	>7 vs ≤7	2.112 (1.349, 3.307)	0.001	1.417 (0.864, 2.321)	0.167
Chemotherapy cycles before BC	>6 vs ≤6	1.344 (0.838, 2.153)	0.220		
Numbers of co-infected locations	≥2 vs <2	2.137 (1.318, 3.465)	0.002	1.468 (0.871, 2.475)	0.150
Fungal BSI	Presence vs Absence	1.099 (0.506, 2.385)	0.812		
G+ bacteria BSI	Presence vs Absence	0.51 (0.282, 0.923)	0.026	1.112 (0.427, 2.895)	0.829
G- bacteria BSI	Presence vs Absence	1.646 (1.001, 2.705)	0.049	1.962 (0.872, 4.416)	0.103
Polymicrobial BSI	Presence vs Absence	3.042 (1.112, 8.321)	0.030	3.44 (1.138, 10.395)	0.029
Polymicrobial infection	≥2 vs <2	1.391 (0.736, 2.627)	0.309		
Present BSI before hospitalization	Done vs Not done	0.981 (0.568, 1.696)	0.946		
Co-infections					
Oral	Presence vs Absence	0.875 (0.528, 1.451)	0.605		
Respiratory	Presence vs Absence	6.651 (2.099, 21.075)	0.001	4.85 (1.49, 15.793)	0.009
Gastrointestinal	Presence vs Absence	1.51 (0.947, 2.407)	0.084		
Skin and soft tissue	Presence vs Absence	2.325 (1.306, 4.137)	0.004	1.489 (0.799, 2.773)	0.210
Perirectal	Presence vs Absence	0.639 (0.294, 1.387)	0.257		
Urinary tract	Presence vs Absence	1.06 (0.335, 3.359)	0.921		
Use of antibiotics, ≥3 agents	Done vs Not done	2.628 (1.144, 6.037)	0.023	1.372 (0.583, 3.229)	0.469

For categorical variables, the coding direction indicates the comparison with the reference category used in regression analyses. The coding strategy was consistent between regression models and nomograms. Abbreviations: AML, acute myeloid leukemia; ALL, acute lymphoblastic leukemia; LOS, length of stay; WBC, white blood cell count; N, neutrophilic count; HB, hemoglobin; PLT, platelet count; PCT, procalcitonin; CRP, C-reactive protein; BC, blood culture; BSI, bloodstream infection; G+, Gram-positive; G-, Gram-negative.

### Calibration and validation of the nomogram for predicting post-BSI mortality

**Figure 5:**
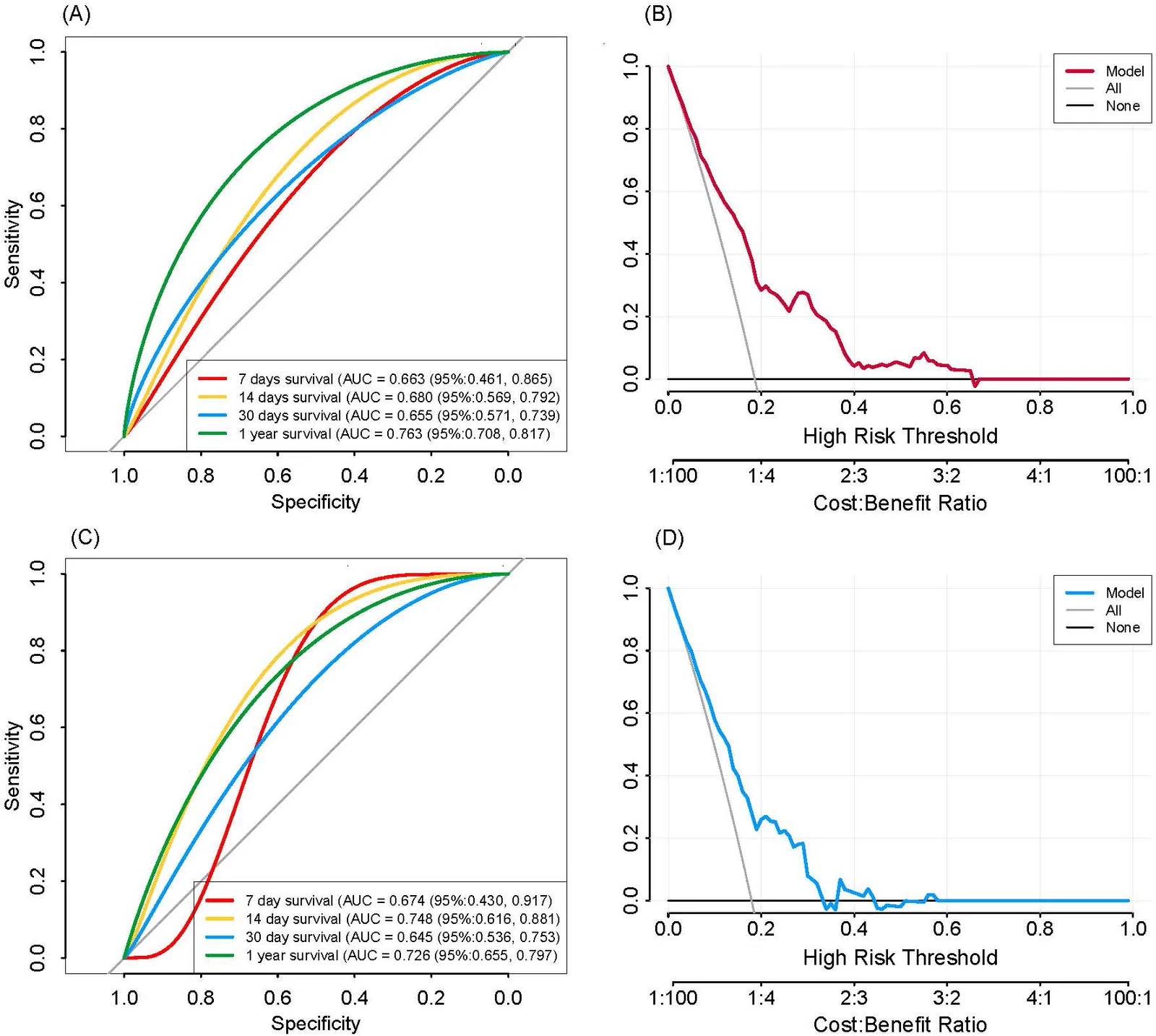
Performance of the post-BSI mortality prediction model in the derivation cohort and internal validation cohort. **(A)** The ROC curve and AUC of the post-BSI mortality prediction model in the derivation cohort. **(B)** The Clinical Decision Curve (CDC) of the post-BSI mortality prediction model in the derivation cohort. The red line was above gray or black line, showed the model provides a higher net benefit. **(C)** The ROC curve and AUC of the post-BSI mortality prediction model in the internal validation cohort. **(D)** The Clinical Decision Curve (CDC) of the post-BSI mortality prediction model in the internal validation cohort. The blue line was above gray or black line, showed the model provides a higher net benefit.

**Figure 6:**
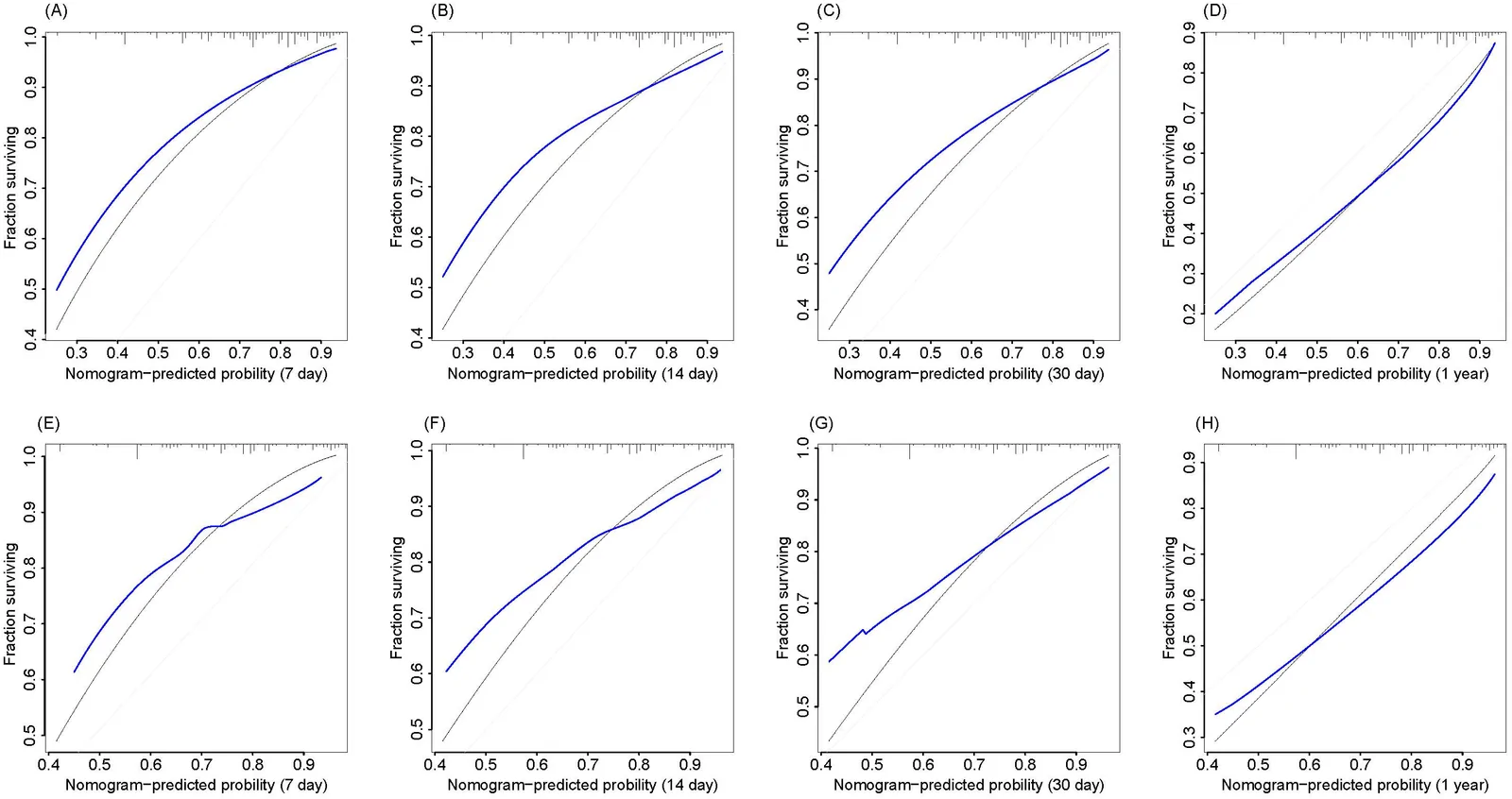
Calibration curve of the post-BSI mortality prediction model at different time points in the derivation cohort and validation cohort. Calibration curve of the nomogram in the derivation cohort **(A–D)**. Calibration curve of the nomogram in the internal validation cohort **(E–H)**. The 45-degree line represents perfect calibration.

Time-dependent ROC was used to assess the predictive performance of the model, which showed good discrimination and accurate all-cause mortality prediction, with a 7-day, 14-day, 30-day and 1-year AUCs in the derivation cohort of 0.66 (95% CI, 0.46-0.87), 0.68 (95% CI, 0.57-0.79), 0.66 (95% CI, 0.57-0.74), and 0.76 (95% CI, 0.71-0.82), respectively (**Figure 5A**). Meanwhile, the 7-day, 14-day, 30-day and 1-year AUCs in the validation cohort of 0.67 (95% CI, 0.43-0.92), 0.75 (95% CI, 0.62-0.88), 0.65 (95% CI, 0.54-0.75), and 0.73 (95% CI, 0.66-0.80), respectively (**Figure 5C**). The mortality model yielded a C-indices of 0.721 (95% CI, 0.68–0.76) in the derivation cohort and 0.713 (95% CI, 0.66–0.76) in the internal validation cohort. The decision curves of the nomogram in the derivation and validation cohorts showed that the model for predicting mortality after BSI was more beneficial than all patients with BSIs or none with BSIs (**Figures 5B, D**). The calibration plot (**Figure 6**) showed an acceptable agreement between the predicted and observed mortality probabilities at the 7-day, 14-day, 30-day and 1-year time point in both the derivation and internal validation cohorts.

### Preliminary assessment of the BSI occurrence model in an independent cohort

**Figure 7:**
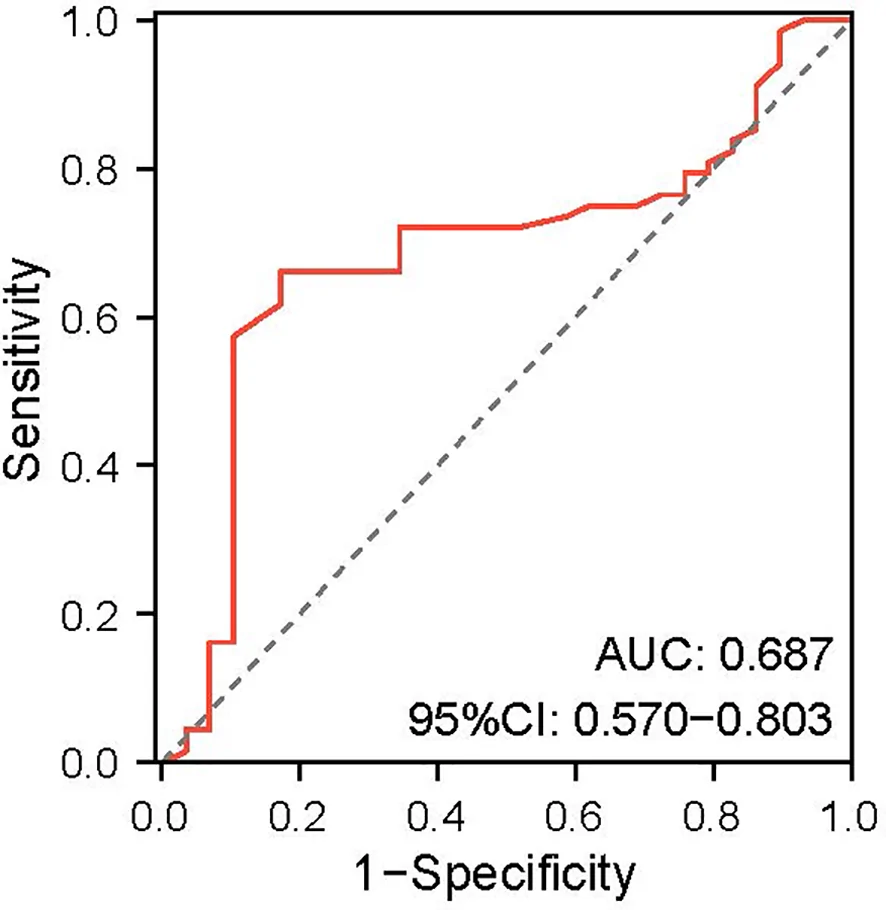
Preliminary assessment of the BSI occurrence model in an independent cohort. The BSI occurrence model was assessed in an independent cohort of 97 patients with acute leukemia undergoing hematopoietic stem cell transplantation. AUC, area under the curve; CI, confidence interval.

An independent cohort of 97 patients (**Supplementary Table 5**) with acute leukemia who subsequently underwent hematopoietic stem cell transplantation was used for a preliminary assessment of the BSI occurrence model. Compared with the derivation cohort, this cohort represented a more selected clinical population with differences in disease composition, treatment setting, and clinical characteristics. During follow-up, 28 patients developed BSI. The BSI occurrence model showed an AUC of 0.687 (95% CI, 0.57–0.80; **Figure 7**) in this independent cohort. Given the limited sample size and the specific characteristics of this cohort, these findings should be interpreted as preliminary evidence supporting the potential transportability of the model rather than definitive external validation across the broader population of patients with hematologic malignancies.

The independent cohort was not used for external performance of the post-BSI mortality model. No deaths occurred within 7, 14, or 30 days among patients with BSI in this cohort, whereas the 1-year outcome distribution was highly imbalanced. This outcome pattern did not permit a meaningful assessment of short-term discrimination, calibration, or risk separation. Therefore, no external performance estimates were calculated for the mortality model.

## Discussion

BSIs are a severe complication in hospitalized patients (Kargaltseva et al., 2022), particularly among those with malignancy, where they are associated with high incidence and mortality rates (Amanati et al., 2021).

Annual nationwide age-standardized incidence of hospitalized sepsis for both male and female markedly increased from 328.25 cases per 100,000 in 2017 to 421.85 in 2019 And, the corresponding standardized in-hospital sepsis mortality rate gradually increased from 17.32 per 100,000 population in 2017 to 19.16 in 2019 (Weng et al., 2023). GNB are the predominant pathogens in BSIs, with *Escherichia coli* consistently reported as the leading causative agent in most settings (Kern and Rieg, 2020). At present, BC is considered the gold standard for diagnosing BSIs (Fabre et al., 2022; Lamy et al., 2020). However, its limitations, including a low detection rate and dependence on clinicians’ assessment of BSI risk, emphasize the critical need for proactive risk assessment and early warning systems in clinical practice. The serious nature of BSIs warrants increased attention and highlights the necessity for expanded research to identify high-risk populations early, with a particular focus on HM patients.

In this study, we developed and internally validated a BSI occurrence nomogram for clinical risk stratification in patients with HMs. This model was not designed as an admission-based prediction tool; instead, it incorporated clinically available variables during blood-culture evaluation. Hospital length of stay represents a dynamic variable reflecting the accumulated inpatient course and should therefore be interpreted within the context of clinical risk stratification rather than baseline prediction. The AUC values in both the derivation and validation cohorts were greater than 0.7, indicating its potential value for identifying patients at higher risk of BSI. Evaluation using the Hosmer-Lemeshow test, calibration plots, and decision curve analysis demonstrated good calibration performance, consistency, and clinical benefit of the nomogram. Additionally, external validation yielded an AUC of 0.687, indicating an acceptable level of model reliability. Additionally, we developed a post-BSI mortality risk prediction model using data from the BSI subgroup of HM patients. Similarly, analysis with ROC curves, C-index, calibration plots, and clinical decision curves demonstrated that the model has good predictive performance.

Patients with hematologic disorders undergoing chemotherapy, transplantation, or targeted therapy often experience neutropenia, which increases their susceptibility to infections, particularly making them highly vulnerable to BSIs (Erdem et al., 2023; Peri et al., 2023; Peseski et al., 2021). Neutrophil count contributed substantially to the BSI model, which is biologically plausible because neutrophils are central to innate antimicrobial defense and profound neutropenia increases susceptibility to invasive infection. However, the model estimates associations for prediction and do not establish that modifying neutrophil counts will reduce BSI risk. Understanding these mechanisms is crucial for developing effective strategies to prevent and manage BSIs in neutropenic patients. Decisions regarding granulocyte colony-stimulating factor (Abdel-Azim et al., 2019) and empirical antimicrobial therapy (Rhee et al., 2020; Schmidt-Hieber et al., 2019) should therefore follow established clinical indications rather than the nomogram alone.

Polymicrobial BSIs were associated with an increased 90-day mortality rate (OR, 2.20; 95% CI, 1.98-2.60) (Yo et al., 2019). In other studies that have developed prognostic models for BSIs (De la Rosa-Riestra et al., 2024; Ong et al., 2024), polymicrobial infection is also identified as an important high-risk factor. Among the variables retained in our final mortality model, polymicrobial BSI contributed a relatively high point value in the nomogram. The point allocation reflects the contribution of each variable within the fitted model and should not be interpreted as evidence of a causal or independently established effect. The number of polymicrobial BSI episodes was small, resulting in uncertainty regarding the stability of its estimated contribution to the post-BSI mortality prediction model. The apparent contribution of this variable may therefore be unstable and requires confirmation in larger independent cohorts. Many multivariate models predicting BSI probability have been published, but they often suffer from limited applicability, poor discrimination and calibration, and significant inter-model disagreement. Notable discrepancies in expected BSI probabilities were observed between models, with a median (25th-75th percentile) relative difference of 68.0% (28.6-113.6%) between expected risks (Rodic et al., 2023). A acceptable prediction model should ideally possess high sensitivity and a high negative predictive value; however, the accuracy of many existing models is often questioned. The post-BSI mortality model developed in this study achieved an AUC greater than 0.6, indicating adequate discriminative ability. Nonetheless, the inclusion of multiple factors in the model affects its precision. Evaluating the sensitivity, specificity, positive predictive value, and negative predictive value for the 7-day, 14-day, 30-day, and 1-year survival prediction models (**Supplementary Tables 6**, **7**), we found that in both the derivation and validation cohorts, the sensitivity for predicting 14-day survival exceeded 0.80, with positive predictive values above 0.70 and negative predictive values of 0.63 and 0.80, respectively. These results suggest that the model performs well in predicting 14-day survival, thereby supporting short-term clinical decision-making.

This study developed a BSI risk stratification and prognosis model using real-world clinical data from patients with hematologic malignancies. Our cohort was collected between 2013 and 2016. Changes in antimicrobial resistance patterns, infection-prevention strategies, supportive care, and treatment approaches may limit the applicability of the models to contemporary populations. Further validation in recent and geographically diverse cohorts is required. The model integrates demographic factors such as age and gender, alongside disease-specific factors including neutropenia, the number of chemotherapy cycles, and disease status. Additionally, it incorporates commonly used inflammatory markers, PCT and CRP, to improve the model’s clinical applicability.

This study has several limitations: (1) Clinical data were retrospectively collected and analyzed, resulting in missing information, such as data on sequential organ failure assessment, systemic inflammatory response syndrome, and albumin levels. Consequently, these factors were not included in the analysis; (2) The independent cohort was small and consisted exclusively of patients with acute leukemia who underwent hematopoietic stem cell transplantation. This population differed from the heterogeneous derivation cohort in disease spectrum, age distribution, treatment intensity, and clinical course. Consequently, spectrum bias may have affected model performance, and the findings from this cohort should not be interpreted as evidence of general applicability to all patients with hematologic malignancies. The external analysis provides only preliminary support for the transportability of the BSI occurrence model. (3) The post-BSI mortality model was evaluated in the derivation and internal validation cohorts only. The independent cohort contained a limited number of mortality events with an imbalanced distribution, limiting the feasibility of a reliable external assessment of this model. Therefore, the transportability of the post-BSI mortality model to other patient populations requires further evaluation in larger and more representative cohorts with adequate mortality events.

In summary, we developed and internally validated nomograms for predicting BSI occurrence and post-BSI mortality among patients with HMs. The BSI occurrence model showed acceptable discrimination in the derivation and internal validation cohorts and was preliminarily assessed in an independent cohort. The post-BSI mortality model demonstrated moderate predictive performance in the internal validation cohort, while its transportability requires further evaluation in larger and more representative populations. These models may serve as potential tools for clinical risk stratification and support individualized decision-making for patients with HMs.

## Data Availability

The original contributions presented in the study are included in the article/**Supplementary Material**. Further inquiries can be directed to the corresponding authors.

## Supplementary material

Supplementary Figure 1 Study flow diagram and analytical populations. The overall cohort consisted of 3,014 patients with hematologic malignancies who underwent blood culture evaluation between 2013 and 2016, including 725 patients with confirmed bloodstream infection (BSI) and 2,289 patients without BSI. The BSI occurrence prediction model was developed using the entire cohort and randomly allocated into a derivation cohort (n=1,809) and an internal validation cohort (n=1,205) at a 6:4 ratio. The post-BSI mortality prediction model was developed specifically among patients with confirmed BSI (n=725), which were randomly allocated into a derivation cohort (n=435) and an internal validation cohort (n=290) at a 6:4 ratio. A selected independent hematopoietic stem cell transplantation (HSCT) cohort (n=97; BSI:28, No BSI:69) was used for exploratory evaluation of the BSI occurrence prediction model and was not included in the development or validation of the post-BSI mortality prediction model.
