# Supplementary material for: A multidimensional nomogram for clinical risk stratification of bloodstream infection and mortality in hematologic malignancies
Source: Front Cell Infect Microbiol. 2026 Aug 27;16:1834653. doi: 10.3389/fcimb.2026.1834653 (PMC13558008; doi:10.3389/fcimb.2026.1834653)
Supplement: Supplementary file 1 [file Table1.docx]

Supplementary TableS1 Clinical characteristics of 3014 patients in the derivation cohort and validation cohort

| Variable | Overall, No.(%) n=3014 | Derivation cohort, No.(%) n=1809 | Validation cohort, No.(%) n=1205 | P-value |
| --- | --- | --- | --- | --- |
| BSI |  |  |  |  |
| Absent | 2289 (75.9) | 1380 (76.3) | 909 (75.4) | 0.623 |
| Present | 725 (24.1) | 429 (23.7) | 296 (24.6) |  |
| Gender |  |  |  |  |
| Female | 1322 (43.9) | 823 (45.5) | 499 (41.4) | 0.030 |
| Male | 1692 (56.1) | 986 (54.5) | 706 (58.6) |  |
| Age |  |  |  |  |
| ≤60 | 2505 (83.1) | 1522 (84.1) | 983 (81.6) | 0.074 |
| >60 | 509 (16.9) | 287 (15.9) | 222 (18.4) |  |
| Diagnosis with AML |  |  |  |  |
| No | 1474 (48.9) | 896 (49.5) | 578 (48.0) | 0.421 |
| Yes | 1540 (51.1) | 913 (50.5) | 627 (52.0) |  |
| Diagnosis with ALL |  |  |  |  |
| No | 2450 (81.3) | 1460 (80.7) | 990 (82.2) | 0.341 |
| Yes | 564 (18.7) | 349 (19.3) | 215 (17.8) |  |
| Disease status |  |  |  |  |
| Remission | 640 (21.2) | 395 (21.8) | 245 (20.3) | 0.346 |
| No remission | 2374 (78.8) | 1414 (78.2) | 960 (79.7) |  |
| Hospital LOS, days |  |  |  |  |
| ≤14 | 1230 (40.8) | 736 (40.7) | 494 (41.0) | 0.895 |
| >14 | 1784 (59.2) | 1073 (59.3) | 711 (59.0) |  |
| *WBC, 10^9/L |  |  |  |  |
| ≤4 | 2213 (73.5) | 1319 (73.0) | 894 (74.3) | 0.471 |
| 4~10 | 421 (14.0) | 264 (14.6) | 157 (13.0) |  |
| >10 | 376 (12.5) | 223 (12.3) | 153 (12.7) |  |
| *N, 10^9/L |  |  |  |  |
| ≤0.5 | 1844 (61.3) | 1100 (61.0) | 744 (61.8) | 0.903 |
| 0.5~1.5 | 288 (9.6) | 174 (9.6) | 114 (9.5) |  |
| >1.5 | 876 (29.1) | 530 (29.4) | 346 (28.7) |  |
| *HB, g/L |  |  |  |  |
| ≤60 | 1126 (37.4) | 672 (37.2) | 454 (37.7) | 0.812 |
| >60 | 1884 (62.6) | 1134 (62.8) | 750 (62.3) |  |
| *PLT, 10^9/L |  |  |  |  |
| ≤20 | 1262 (41.9) | 770 (42.6) | 492 (40.9) | 0.354 |
| >20 | 1748 (58.1) | 1036 (57.4) | 712 (59.1) |  |
| PCT, ng/ml |  |  |  |  |
| ≤0.24 | 1515 (50.3) | 909 (50.2) | 606 (50.3) | 1.000 |
| >0.24 | 1499 (49.7) | 900 (49.8) | 599 (49.7) |  |
| Duration of neutropenia before BC, days | | |  |  |
| ≤7 | 2577 (85.5) | 1547 (85.5) | 1030 (85.5) | 1.000 |
| >7 | 437 (14.5) | 262 (14.5) | 175 (14.5) |  |
| Chemotherapy cycles before BC | |  |  |  |
| ≤6 | 2427 (80.5) | 1459 (80.7) | 968 (80.3) | 0.865 |
| >6 | 587 (19.5) | 350 (19.3) | 237 (19.7) |  |
| Numbers of co-infected locations | |  |  |  |
| <2 | 2718 (90.2) | 1637 (90.5) | 1081 (89.7) | 0.519 |
| ≥2 | 296 (9.8) | 172 (9.5) | 124 (10.3) |  |
| Co-infections |  |  |  |  |
| Oral |  |  |  |  |
| Absence | 2405 (79.8) | 1448 (80.0) | 957 (79.4) | 0.710 |
| Presence | 609 (20.2) | 361 (20.0) | 248 (20.6) |  |
| Respiratory |  |  |  |  |
| Absence | 590 (19.6) | 368 (20.3) | 222 (18.4) | 0.210 |
| Presence | 2424 (80.4) | 1441 (79.7) | 983 (81.6) |  |
| Gastrointestinal |  |  |  |  |
| Absence | 2509 (83.2) | 1500 (82.9) | 1009 (83.7) | 0.591 |
| Presence | 505 (16.8) | 309 (17.1) | 196 (16.3) |  |
| Skin and soft tissue |  |  |  |  |
| Absence | 2776 (92.1) | 1662 (91.9) | 1114 (92.4) | 0.615 |
| Presence | 238 (7.9) | 147 (8.1) | 91 (7.6) |  |
| Perirectal |  |  |  |  |
| Absence | 2737 (90.8) | 1641 (90.7) | 1096 (91.0) | 0.873 |
| Presence | 277 (9.2) | 168 (9.3) | 109 (9.0) |  |
| Urinary tract |  |  |  |  |
| Absence | 2938 (97.5) | 1764 (97.5) | 1174 (97.4) | 0.978 |
| Presence | 76 (2.5) | 45 (2.5) | 31 (2.6) |  |
| Other |  |  |  |  |
| Absence | 2941 (97.6) | 1761 (97.3) | 1180 (97.9) | 0.373 |
| Presence | 73 (2.4) | 48 (2.7) | 25 (2.1) |  |
| Diabetes mellitus |  |  |  |  |
| Absence | 2710 (89.9) | 1619 (89.5) | 1091 (90.5) | 0.385 |
| Presence | 304 (10.1) | 190 (10.5) | 114 (9.5) |  |
| Use of antibiotics, ≥3 agents | |  |  |  |
| Not done | 888 (29.5) | 549 (30.3) | 339 (28.1) | 0.205 |
| Done | 2126 (70.5) | 1260 (69.7) | 866 (71.9) |  |

Annotation: abbreviations: BSI, bloodstream infection; AML, acute myeloid leukemia; ALL, acute lymphoblastic leukemia; LOS, length of stay; WBC, white blood cell count; N, neutrophilic count; HB, hemoglobin; PLT, platelet count; PCT, procalcitonin; BC, blood culture. *: There were 4 patients with missing data for WBC, HB, and PLT within 24 hours of BC submission and 6 patients with missing data for N.

Supplementary TableS2 Clinical characteristics of patients included in the post-BSI mortality prediction model

| Variable | Overall, No.(%) n=725 | Derivation cohort, No.(%) n=435 | Validation cohort, No.(%) n=290 | P-value |
| --- | --- | --- | --- | --- |
| Gender |  |  |  |  |
| Male | 435 (60.0) | 263 (60.5) | 172 (59.3) | 0.816 |
| Female | 290 (40.0) | 172 (39.5) | 118 (40.7) |  |
| Age |  |  |  |  |
| ≤60 | 633 (87.3) | 382 (87.8) | 251 (86.6) | 0.699 |
| >60 | 92 (12.7) | 53 (12.2) | 39 (13.4) |  |
| Diagnosis with AML |  |  |  |  |
| No | 377 (52.0) | 233 (53.6) | 144 (49.7) | 0.339 |
| Yes | 348 (48.0) | 202 (46.4) | 146 (50.3) |  |
| Diagnosis with ALL |  |  |  |  |
| No | 513 (70.8) | 303 (69.7) | 210 (72.4) | 0.474 |
| Yes | 212 (29.2) | 132 (30.3) | 80 (27.6) |  |
| Disease status |  |  |  |  |
| Remission | 155 (21.4) | 98 (22.5) | 57 (19.7) | 0.405 |
| No remission | 570 (78.6) | 337 (77.5) | 233 (80.3) |  |
| Diabetes mellitus |  |  |  |  |
| Absence | 648 (89.4) | 391 (89.9) | 257 (88.6) | 0.676 |
| Presence | 77 (10.6) | 44 (10.1) | 33 (11.4) |  |
| Hospital LOS, days |  |  |  |  |
| ≤14 | 213 (29.4) | 120 (27.6) | 93 (32.1) | 0.224 |
| >14 | 512 (70.6) | 315 (72.4) | 197 (67.9) |  |
| WBC, 10^9/L |  |  |  |  |
| ≤4 | 652 (89.9) | 393 (90.3) | 259 (89.3) | 0.807 |
| 4~10 | 40 (5.5) | 24 (5.5) | 16 (5.5) |  |
| >10 | 33 (4.6) | 18 (4.1) | 15 (5.2) |  |
| N, 10^9/L |  |  |  |  |
| ≤0.5 | 622 (85.8) | 382 (87.8) | 240 (82.8) | 0.110 |
| 0.5~1.5 | 33 (4.6) | 19 (4.4) | 14 (4.8) |  |
| >1.5 | 70 (9.7) | 34 (7.8) | 36 (12.4) |  |
| HB, g/L |  |  |  |  |
| ≤60 | 346 (47.7) | 222 (51.0) | 124 (42.8) | 0.035 |
| >60 | 379 (52.3) | 213 (49.0) | 166 (57.2) |  |
| PLT, 10^9/L |  |  |  |  |
| ≤20 | 428 (59.0) | 265 (60.9) | 163 (56.2) | 0.235 |
| >20 | 297 (41.0) | 170 (39.1) | 127 (43.8) |  |
| PCT, ng/ml |  |  |  |  |
| ≤0.6 | 363 (50.1) | 223 (51.3) | 140 (48.3) | 0.476 |
| >0.6 | 362 (49.9) | 212 (48.7) | 150 (51.7) |  |
| CRP, mg/L |  |  |  |  |
| ≤92.9 | 364 (50.2) | 213 (49.0) | 151 (52.1) | 0.458 |
| >92.9 | 361 (49.8) | 222 (51.0) | 139 (47.9) |  |
| Duration of neutropenia before BC, days | |  |  |  |
| ≤7 | 547 (75.4) | 330 (75.9) | 217 (74.8) | 0.819 |
| >7 | 178 (24.6) | 105 (24.1) | 73 (25.2) |  |
| Chemotherapy cycles before BC | |  |  |  |
| ≤6 | 553 (76.3) | 325 (74.7) | 228 (78.6) | 0.262 |
| >6 | 172 (23.7) | 110 (25.3) | 62 (21.4) |  |
| Numbers of co-infected locations | |  |  |  |
| <2 | 605 (83.4) | 364 (83.7) | 241 (83.1) | 0.919 |
| ≥2 | 120 (16.6) | 71 (16.3) | 49 (16.9) |  |
| Fungal BSI |  |  |  |  |
| Absence | 669 (92.3) | 401 (92.2) | 268 (92.4) | 1.000 |
| Presence | 56 (7.7) | 34 (7.8) | 22 (7.6) |  |
| G+ bacteria BSI |  |  |  |  |
| Absence | 527 (72.7) | 324 (74.5) | 203 (70.0) | 0.214 |
| Presence | 198 (27.3) | 111 (25.5) | 87 (30.0) |  |
| G- bacteria BSI |  |  |  |  |
| Absence | 262 (36.1) | 151 (34.7) | 111 (38.3) | 0.368 |
| Presence | 463 (63.9) | 284 (65.3) | 179 (61.7) |  |
| Polymicrobial BSI |  |  |  |  |
| Absence | 708 (97.7) | 425 (97.7) | 283 (97.6) | 1.000 |
| Presence | 17 (2.3) | 10 (2.3) | 7 (2.4) |  |
| Polymicrobial infection |  |  |  |  |
| <2 | 646 (89.1) | 390 (89.7) | 256 (88.3) | 0.644 |
| ≥2 | 79 (10.9) | 45 (10.3) | 34 (11.7) |  |
| Present BSI before hospitalization | |  |  |  |
| Not done | 586 (80.8) | 348 (80.0) | 238 (82.1) | 0.551 |
| Done | 139 (19.2) | 87 (20.0) | 52 (17.9) |  |
| Co-infections |  |  |  |  |
| Oral |  |  |  |  |
| Absence | 545 (75.2) | 319 (73.3) | 226 (77.9) | 0.188 |
| Presence | 180 (24.8) | 116 (26.7) | 64 (22.1) |  |
| Respiratory |  |  |  |  |
| Absence | 139 (19.2) | 81 (18.6) | 58 (20.0) | 0.714 |
| Presence | 586 (80.8) | 354 (81.4) | 232 (80.0) |  |
| Gastrointestinal |  |  |  |  |
| Absence | 550 (75.9) | 329 (75.6) | 221 (76.2) | 0.929 |
| Presence | 175 (24.1) | 106 (24.4) | 69 (23.8) |  |
| Skin and soft tissue |  |  |  |  |
| Absence | 658 (90.8) | 396 (91.0) | 262 (90.3) | 0.855 |
| Presence | 67 (9.2) | 39 (9.0) | 28 (9.7) |  |
| Perirectal |  |  |  |  |
| Absence | 628 (86.6) | 381 (87.6) | 247 (85.2) | 0.410 |
| Presence | 97 (13.4) | 54 (12.4) | 43 (14.8) |  |
| Urinary tract |  |  |  |  |
| Absence | 693 (95.6) | 420 (96.6) | 273 (94.1) | 0.172 |
| Presence | 32 (4.4) | 15 (3.4) | 17 (5.9) |  |
| Use of antibiotics |  |  |  |  |
| ≥2 agents |  |  |  |  |
| Not done | 16 (2.2) | 8 (1.8) | 8 (2.8) | 0.570 |
| Done | 709 (97.8) | 427 (98.2) | 282 (97.2) |  |
| ≥3 agents |  |  |  |  |
| Not done | 120 (16.6) | 72 (16.6) | 48 (16.6) | 1.000 |
| Done | 605 (83.4) | 363 (83.4) | 242 (83.4) |  |

Annotation: abbreviations: AML, acute myeloid leukemia; ALL, acute lymphoblastic leukemia; LOS, length of stay; WBC, white blood cell count; N, neutrophilic count; HB, hemoglobin; PLT, platelet count; PCT, procalcitonin; CRP, C-reactive protein; BC, blood culture; BSI, bloodstream infection; G+, Gram-positive; G-, Gram-negative.

Supplementary Table 3 Full specification of the BSI occurrence prediction model

| **Predictor** | **Coding** | **β coefficient** |
| --- | --- | --- |
| Gender | Male vs Female | 0.431 |
| Age | >60 vs ≤60 | -0.289 |
| WBC (10⁹/L) | 4–10 vs ≤4 | 0.192 |
| WBC (10⁹/L) | >10 vs ≤4 | -0.732 |
| Neutrophil count (10⁹/L) | 0.5–1.5 vs ≤0.5 | -1.13 |
| Neutrophil count (10⁹/L) | >1.5 vs ≤0.5 | -1.241 |
| Platelet count (10⁹/L) | >20 vs ≤20 | -0.476 |
| Hospital LOS, days | >14 vs ≤14 | 0.552 |
| Chemotherapy cycles before BC | >6 vs ≤6 | 0.417 |
| Number of co-infected locations | ≥2 vs <2 | 0.392 |
| PCT,ng/mL | >0.24 vs ≤ 0.24 | 0.952 |
| Diagnosis of ALL | Yes vs No | 0.379 |
| Urinary tract infection | Presence vs Absence | 0.721 |
| Antibiotic use ≥3 agents | Yes vs No | 0.473 |

Annotation: β coefficients were obtained from the final multivariable logistic regression model. Categorical predictors were coded according to the reference categories shown in the Table1. The coding strategy was consistently applied in regression analyses and nomogram construction. BSI, bloodstream infection; BC, blood culture; LOS, length of stay; PCT, procalcitonin; ALL, acute lymphoblastic leukemia.

Supplementary TableS4 Full specification of the final post-BSI mortality prediction model

| **Predictor** | **Coding** | **β coefficient** |
| --- | --- | --- |
| Duration of neutropenia before BC | >7 days vs ≤7 days | 0.349 |
| HB, g/L | >60 vs ≤60 | -0.524 |
| PCT, ng/mL | >0.6 vs ≤0.6 | 0.882 |
| CRP, mg/L | >92.9 vs ≤92.9 | 0.329 |
| Number of co-infected locations | ≥2 vs <2 | 0.384 |
| G- bacteria BSI | Presence vs Absence | 0.674 |
| Polymicrobial BSI | Presence vs Absence | 1.235 |
| Disease status | Non-remission vs Remission | 0.556 |
| Respiratory infection | Presence vs Absence | 1.579 |

Annotation: β coefficients were obtained from the final multivariable Cox proportional hazards regression model. Categorical predictors were coded according to the reference categories shown in the Table2. The coding strategy was consistently applied in regression analyses and nomogram construction. BSI, bloodstream infection; BC, blood culture; PCT, procalcitonin; CRP, C-reactive protein.

Supplementary TableS5 Clinical characteristics of 97 acute leukemia patients.

| **Variables** | **No.(%)** |
| --- | --- |
| Diagnosis |  |
| AML | 54(55.7) |
| ALL | 38(39.2) |
| Others | 5(5.1) |
| Gender |  |
| Male | 56(57.7) |
| Female | 41(42.3) |
| Age, median(range), y | 35.0(14.0-62.0) |
| BSI |  |
| Present | 28(28.9) |
| Absent | 69(71.1) |
| Outcome of BSI patients |  |
| 14-day mortality | 0(0.0) |
| 30-day mortality | 0(0.0) |
| 1-year mortality | 27(96.4%) |

Abbreviation: BSI, bloodstream infection; AML, acute myeloid leukemia; ALL, acute lymphoblastic leukemia; G+, Gram-positive; G-, Gram-negative.

Supplementary TableS6 Receiver operating characteristic curve accuracy metrics for the BSI occurrence prediction model.

| **BSI occurrence prediction model** | **Specificity** | **Sensitivity** | **NPV** | **PPV** |
| --- | --- | --- | --- | --- |
| Derivation cohort | 0.69 | 0.72 | 0.88 | 0.43 |
| Validation cohort | 0.79 | 0.59 | 0.86 | 0.46 |

Abbreviation: NPV, Negative predictive value; PPV, Positive predictive value.

Supplementary TableS7 Receiver operating characteristic curve accuracy metrics for the BSI mortality risk model.

| **BSI mortality risk model** | **Survival time** | **Specificity** | **Sensitivity** | **NPV** | **PPV** |
| --- | --- | --- | --- | --- | --- |
| Derivation cohort | 7-day | 0.56 | 0.76 | 0.28 | 0.91 |
| Derivation cohort | 14-day | 0.51 | 0.82 | 0.63 | 0.75 |
| Derivation cohort | 30-day | 0.76 | 0.52 | 0.61 | 0.69 |
| Derivation cohort | 1-year | 0.59 | 0.81 | 0.93 | 0.31 |
| Validation cohort | 7-day | 0.50 | 1.00 | 1.00 | 0.87 |
| Validation cohort | 14-day | 0.50 | 0.93 | 0.80 | 0.76 |
| Validation cohort | 30-day | 0.41 | 0.87 | 0.74 | 0.61 |
| Validation cohort | 1-year | 0.73 | 0.62 | 0.90 | 0.33 |

Abbreviation: NPV, Negative predictive value; PPV, Positive predictive value.
